# Inflammatory responses and barrier disruption in the trachea of chicks following *Mycoplasma gallisepticum* infection: a focus on the TNF-α-NF-κB/MLCK pathway

**DOI:** 10.1186/s13567-023-01259-6

**Published:** 2024-01-15

**Authors:** Lemiao Zhong, Chunlin Wu, Yan Zhao, Baoqin Huang, Zhongbao Luo, Yijian Wu

**Affiliations:** 1 University Key Laboratory for Integrated Chinese Traditional and Western Veterinary Medicine and Animal Healthcare in Fujian Province, Fuzhou, 350002 China; 2 Fujian Key Laboratory of Traditional Chinese Veterinary Medicine and Animal Health, Fujian Agriculture and Forestry University, Fuzhou, 350002 China; 3 Fujian Sunner Development Co. Ltd., Nanping, 354100 China

**Keywords:** *Mycoplasma gallisepticum*, chicken embryo tracheal organ culture, inflammatory cytokines, NF-κB/MLCK pathway, TJ protein, tracheal epithelial barrier

## Abstract

*Mycoplasma gallisepticum* (MG) can induce persistent inflammatory damage to the tracheal mucosa of poultry and cause chronic respiratory diseases in chickens. To further investigate the mechanism of MG-induced injury to the tracheal mucosa, we used chick embryo tracheal organ culture (TOC) as a model to study the invasion and reproduction of MG, the effect of MG on tracheal morphology, and the potential factors that promote MG tissue invasion. The results showed that MG infection significantly damaged the tracheal epithelial structure and weakened tracheal epithelial barrier function; MG also increased the occurrence of bacterial displacement, with a significant (*p* < 0.05) increase in the bacterial load of the infected TOCs at 5 and 7 days post-infection. In addition, MG significantly (*p* < 0.05) increased the expression levels of inflammatory cytokines, such as TNF-α, interleukin-1β (IL-1β), and IL-6, and activated the NF-κB signalling pathway, leading to increased nuclear translocation of NF-κB p65. Simultaneously, the map kinase pathway (MAPK) was activated. This activation might be associated with increased myosin light chain (MLC) phosphorylation, which could lead to actin-myosin contraction and disruption of tight junction (TJ) protein function, potentially compromising epithelial barrier integrity and further catalysing MG migration into tissues. Overall, our results contribute to a better understanding of the interaction between MG and the host, provide insight into the mechanisms of damage to the tracheal mucosa induced by MG infection, and provide new insights into the possible pathways involved in *Mycoplasma gallisepticum* infection in vivo.

## Introduction

*Mycoplasma gallisepticum* (MG) belongs to the class Mollicutes and family Mycoplasmataceae. MG is highly pathogenic to poultry, especially chickens and turkeys, and causes chronic respiratory disease (CRD). Thus, MG has caused significant economic losses to the poultry industry worldwide [1]. The main site of *Mycoplasma gallisepticum* infection is the tracheal mucosa, and infection leads to epithelial cell degeneration, tracheal epithelial thickening, and inflammatory cell infiltration, which leads to the shedding of respiratory epithelial cells and cilia and tracheal epithelial barrier damage. Although the tracheal inflammatory response induced by *Mycoplasma gallisepticum* infection has been widely studied, MG has also been detected in tissues outside of the respiratory tract, such as the urogenital tract, bile, and brain [2, 3], suggesting that *M. gallisepticum* can translocate across the respiratory mucosal barrier, enter the bloodstream, and disseminate throughout the body [4]. However, to date, the mechanisms underlying the interaction between MG and the tracheal epithelial barrier have not been well described, and how MG crosses the mucosal barrier in chickens remains unclear.

The tracheal epithelial barrier, which is composed of tracheal epithelial cells and associated lymphoid tissues, is generally considered to be the first line of defence against respiratory pathogens [5]. This barrier limits the entry of luminal commensal bacteria and pathogens into the body but is not completely impermeable [6]. Increasing evidence suggests that the tracheal epithelial immune response plays an important role in tracheal barrier function. Beaudet et al. [7] found that inflammation in the trachea was mediated by proinflammatory cytokines and chemokines through Toll-like receptor (TLR) 2 signalling through the NF-κB pathway by examining the transcriptional profiles of tracheal responses in chickens infected with blank or *Mycoplasma gallisepticum* strain Rlow for 7 days. In addition, proinflammatory cytokines such as TNF-α, IFN-γ, and IL-1β are involved in tracheal epithelial barrier dysfunction and lead to the phosphorylation of myosin light chain (MLC) by upregulating the expression and activity of epithelial myosin light chain kinase (MLCK). Studies have shown that for epithelial tight junctions (TJs) to experience increased permeability due to bacterial toxins, pathogens, and Na^+^-glucose cotransporters, it is crucial to activate MLCK followed by contraction of the surrounding actomyosin/myosin filaments. Within these TJ modulation mechanisms, there is swift activation of MLCK, which subsequently increases tracheal TJ permeability. This activation prompts actomyosin to contract, thereby stimulating reshaping of the cell’s structural framework. As a result, tight junction proteins are diminished, thus paving the way for both direct cellular and adjacent cellular pathways that promote the internal proliferation of harmful microorganisms [8, 9].

Tracheal organ cultures (TOC) have been effectively established and are now recognized as a valuable model for studying interactions between viruses and their hosts across various species [10, 11]. This model stands out because it enhances the visibility of signalling molecules and reduces the impact of environmental factors compared to in vivo studies [12]. This model also scores high on the metrics of time efficiency, spatial considerations, and repeatability [13]. Considering these strengths, in our research adopted the TOC approach for an ex vivo infection investigation. As this organ culture method emulates natural infections, this method is pivotal in shedding light on how MG infection damages the tracheal lining in chickens.

In this study, we aimed to construct chicken tracheal organ cultures as an ex vivo model to further investigate the invasion and replication of MG and the mechanisms underlying the consequent tracheal mucosa damage. This encompasses MG activity within the tracheal ring, the formation of tracheal tissue lesions, and the impact of host immune response factors, specifically cytokines and chemokines, on the tight junctions of tracheal epithelial cells. Special emphasis was placed on the regulation of myosin light-chain kinase (MLCK) and tight junction protein (claudin-1, occludin, and ZO-1) expression. This research provides invaluable insights into the mechanisms of the tracheal mucosa damage during MG infection, thereby contributing to the development of prevention and treatment strategies.

## Materials and methods

### Animals and bacteria

The MG-HY strain was isolated, identified, and preserved in our laboratory [14]. SPF eggs were purchased from Jinan (China, Shandong) Spafas Poultry Co., Ltd., and incubated in the laboratory. MG was grown in modified Hayflick medium supplemented with 20% fetal bovine serum (FBS), 10% freshly prepared yeast extract, 0.05% penicillin, 0.05% thallium acetate, and 0.1% nicotinamide adenine dinucleotide (NAD) at 37 °C [15]. The medium colour ranged from phenol red to orange, indicating that MG reached mid-exponential growth at a density of 1 × 10^9^ CCU/mL, which was adjusted for the subsequent experiments [16].

### Preparation of TOCs

TOC was prepared from 18-day-old specific pathogen-free chicken embryos (China, Shandong) as previously described [17]. Briefly, the tracheae were dissected and cleaned from connective tissue and attached muscles and cut with a sterile microtome blade into rings approximately 0.8 mm thick. The tracheal ring was clamped into 37 ℃ prewarmed 199 Hanks medium (Sigma‒Aldrich, Taufkirchen, Bavaria, Germany) containing 1% penicillin/streptomycin (10 000 U/mL, 10 000 mg/mL; Biochrom, Berlin, Germany) and 1% l-glutamine (200 mM; Biochrom) [18, 19]. TOCs were incubated in a 37 ℃ incubator for 48 h. TOC cilia viability was assessed using microscopy, and every ring was divided virtually into 10 parts, with a ciliary activity of 100% assigned to different groups in the experiments.

### Determination of TOC-ID_50_

The tracheal rings showing 100% ciliary activity were clamped into 48-well cell plates with elbow tweezers (1 ring per well) for the control group (*n* = 8) and seven experimental groups (*n* = 8). All experiments were performed in triplicate. The MG-HY strain was diluted tenfold with 199 Hanks’ medium and subsequently added to the seven experimental groups according to the dilutions. The corresponding dilutions (200 µL) were added to each well, and the rings of the control group were incubated with 200 µL of phosphate-buffered saline (PBS). The 48-well plates were incubated at 37 °C and 5% CO_2_ in a cell culture incubator, and the lesions were observed every 12 h (until 144 h) after the tracheal rings were infected with MG. The corresponding experimental results were recorded. The TOC-ID_50_ was calculated according to the Reed–Muench method [20]. According to the degree of lesions in the tissue culture of tracheal rings, the pathological changes in tracheal rings were classified into grades, and the number of tracheal rings with grade 3 lesions was calculated to determine the TOC-ID_50_. The level assessment criteria are listed in Table 1.

**Table 1:** **Evaluation criteria for pathological changes in the tracheal ring**

Histopathological scores	Histopathological changes in tracheal rings
0 (no lesion)	Normal cilia movement around the tracheal ring tissue culture, no shedding of epithelial cells
1 (mild)	50–95% of cilia around the tracheal ring tissue culture stop moving, epithelial cells do not shed
2 (moderate)	Tracheal ring tissue cultures with more than 95% cilia cessation of motility around them and incomplete shedding of epithelial cells
3 (severe)	Tracheal ring tissue cultures without a single ciliary movement around the perimeter, with epithelial cell shedding

### Establishment of an in vitro infection model

The preparation of tracheal organ cultures (TOC) has been previously described. The 48 rings of the trachea were set to 100% cilia activity in 48-well plates (1 ring per well) containing 199 Hanks salt medium (supplemented with 1% l-glutamine [Biochrom, Berlin, Germany], penicillin [1650 U/mg], and 0.2% bovine serum albumin (BSA) (Carl Roth^®^, Karlsruhe, Germany) (600 µL). The patients were randomly assigned to the control or infected group (*n* = 24). According to the TOC-ID_50_ value determined by the Reed-Muench method, the MG-HY strain solution was diluted with 199 Hanks’ salt culture solution. Diluted bacterial solution was added to the infected group at 200 µL/well, and the rings of the control group were incubated with 200 µL of PBS. Both groups were incubated at 37 °C in a 5% CO_2_ incubator, the lesions were observed every 12 h post-infection with MG (observed for 144 h), and the corresponding experimental results were recorded. Tracheal rings were collected at 3, 5 and 7 days post-infection (dpi) (*n* = 8/time points/group), and some of the tracheal tissue was fixed in 4% paraformaldehyde, while the remaining tracheal samples were stored at −80 °C for further experimental analyses.

### Mycoplasma load measurement and FISH assay

To assess the degree of MG infection, we extracted bacterial DNA from the TOC using a kit from Omega Bio-Tek, Inc., Georgia, USA. Two microlitres of extracted sample DNA was added, for a total reaction volume of 20 µL. Amplification reactions were performed using the 7900HT Fast Real-Time PCR System (Life Technologies, Grand Island, NY); PCRs were run for 35 cycles, and the fluorescence of each cycle was monitored at 72 ℃. After amplification, melting curve analysis was performed starting at 60 °C with a temperature shift rate of 0.2 ℃/s. The MG 16S gene (ID: NC_017502.1) was used as the MG genome amplification sequence for the following genes: MG16sRF (5′-GAG CTA ATC TGT AAA GTT GGT C-3′, Tm = 57.80 °C) and MG16sRR (5′-GCT TCC TTG CGG TTA GCA AC-3′, Tm = 63.64 °C). The DNA copies of MG in the chick trachea were detected via qRT‒PCR using a Roche LightCycler instrument (Roche, Shanghai, China). The DNA standard curve was plotted based on cycle values (Ct) and the amount of MG in culture [21].

For fluorescence in situ hybridization, tracheal rings (*n* = 8) were first fixed in 4% paraformaldehyde for 36 h. Following graded dehydration, the samples were cleared, soaked in wax, and embedded. Approximately 3 µm thick tissue sections were then cut, deparaffinized in xylene, and treated with 3% methanol-H_2_O_2_ dropwise to block endogenous peroxidase. The sections were then incubated with 20 µL of hybridization buffer and 15 µg of probe at 37 °C for 16 h in the dark [22]. In this study, the hybridization probes LmCq and 5′-TGC GAA TGT ACT ACT CAG GCA GGA TGT TTA ATG TG (TTT CAT CAT CAT ACA TCA TCA T) were used to detect MG, which was subsequently labelled with Cy3. The samples were then washed in prewarmed hybridization buffer (50% formamide, 50% 2 × SSC) at 37 °C for 15 min, rinsed in distilled water, and air-dried. Images were acquired using a fluorescence microscope (Nikon, Tokyo, Japan) with a specific filter.

### Histopathological examination

The TOC was fixed in 4% formaldehyde and embedded in paraffin. The tissue was excised to a thickness of approximately 3 µm and then stained with haematoxylin and eosin (H&E). The histopathological criteria are based on the integrity of the tracheal mucosa, hyperplasia, submucosal oedema, and cilia loss. The method used was adapted from Gates et al. [23, 24]. The scores were defined as follows: (0) the trachea was intact without lesions; (1) slight mucosal thickening caused by mild diffuse lymphocyte infiltration; (2) diffuse lymphocytes extended from the subepithelial space to the lamina propria; and (3) more than half of the villous epithelium was shed, the villi and lamina propria were exposed, and the tracheal mucosa was necrotic and shed. Blinded provincial evaluation was performed by 3 pathologists.

### Total RNA extraction and qRT‒PCR

To determine the mRNA expression levels of inflammatory factors and tight junction proteins, total RNA was isolated from TOCs using Trizol reagent (Sangon, Shanghai, Beijing) according to the manufacturer’s instructions (Takara Biomedical Technology (Beijing) Co., Ltd., China). The RNA quality was assessed using a Qubit 2.0 RNA kit (Thermo, Shanghai, China), and the total RNA was reverse transcribed to cDNA using a PrimeScript™ RT Master Mix kit (TaKaRa, Dalian, China). The primers used for amplification by qRT‒PCR were gene-specific primers; the primer sequences are listed in Table 2. The qRT‒PCR conditions were as follows: 95 °C–3 min, 95 °C–15 s, 60 °C–30 s, and 72 °C–60 s for 40 cycles [24]. β-actin was used as an internal standard, and the data were quantified using the 2^−ΔΔCt^ method.

**Table 2:** **List of primers used in qRT‒PCR**

Genes	Accession number	Primers (from 5′ to 3′)	Product length (bp)
TNF-α	NM_204267.1	F:TGATCGTGACACGTCTCTGC R:CAACCAGCTATGCACCCCAG	88
IL4	NM_001007079.1	F:GAGCCAGCACTGCCACAAGA R:CCTGCTGCCGTGGGACAT	105
IL-6	NM_204628.1	F:TTCACCGTGTGCGAGAACAGC R:CAGCCGTCCTCCTCCGTCAC	80
IL-8	AJ009800	F: ATGAACGGCAAGCTTGGAGCT R: TCACAGTGGTGCATCAGAATTGA	238
IFN-γ	Y07922	F:GCCGCACATCAAACACATATCT R:CAGTAGGAGGTATAAATACTTTC	302
IL1β	Y15006	F:GGCTCAACATTGCGCTGTAC R:CCCACTTAGCTTGTAGGTGGC	270
CXCL14	NM_204712.2	F:GAACCCCAAACGCCAGAA R:GAGCCCAGTCCTACGTCAGC	138
iNOS2	U46504	F:AGGCCAAACATCCTGGAGGTC R:TCATAGAGACGCTGCTGCCAG	285

### Western blot analysis

Western blotting was performed following a previously described procedure with some modifications [25]. The collected TOCs were lysed using RIPA lysis buffer containing PMSF (Meilunbio, Dalian, China). After incubating on ice for 30 min, the lysate was centrifuged at 10 000 × *g* for 30 min at 4 °C. The protein concentration was determined using a BCA Protein Assay Kit (Beyotime). Equal amounts of proteins (40 µg) were subjected to 8–12% SDS‒PAGE, and the separated proteins were subsequently transferred to PVDF membranes, after which the proteins were blocked with a closure solution (5% skim milk powder) for 2 h at room temperature. The following primary antibodies were used: IκBα (10268-1-AP; Proteintech), p-IκBα (bs-2513R; Bioss), p65 (bs-0465R; Bioss), p-p65 (bs-0982R; Bioss), TNF-α (bsm-33207 M; Bioss), MLCK (ab232949; Abcam), MLC2 (3672; Cell Signaling Technology), P-MLC2 (3672; Cell Signaling Technology), and β-actin (bs-0465R; Bioss). The membranes were incubated with horseradish peroxidase-conjugated secondary antibodies for 2 h at room temperature. Blots were assessed using BeyoECL Plus (Beyotime). The results were analysed using ImageJ (version 1.4.3.67) software [26].

### Immunohistochemistry

Dried paraffin sections were dewaxed using xylene, rehydrated through an ethanol gradient, subjected to antigen retrieval with citrate buffer, and subsequently cooled to room temperature. Then, the cells were incubated in a 3% hydrogen peroxide solution for 10 min to inactivate endogenous enzymes. Nonspecific staining was blocked by incubation with 10% normal goat serum for 15 min. Primary antibodies (Claudin-1, 1:100; purchased from Abcam, USA; Occludin, 1:150; purchased from Booysen, China; ZO-1, 1:200; purchased from Booysen, China) were added and the samples were incubated overnight at 4 °C, after which the tissue sections were incubated with HRP-coupled secondary antibodies for 30 min in a 37 °C incubator. Finally, diaminobenzidine (DAB), which was used as a chromogen to generate the signal, was used for staining and the samples were analysed by light microscopy (BDS400, OPTEC, Chongqing, China). Integrated optical density (IOD) values were quantified using Image-Pro Plus 6.0 (Media Cybernetics, Bethesda, MD, USA).

### Statistical analysis

All experiments were performed at least three times unless otherwise stated, and the data are expressed as the mean ± standard deviation (mean ± SD). Statistical analysis was performed using one-way ANOVA followed by Duncan’s multiple comparisons test in SPSS (SPSS, Chicago, IL) software (version 21.0) and GraphPad software (San Diego, CA, USA) (version 6.01). *P* < 0.05 was considered to indicate statistical significance.

## Results

### Measurement of the TOC-ID_50_

To ensure that the dose of bacteria inoculated with TOCs was consistent, the MG bacterial suspension that had been passaged three times was purified, diluted to seven different concentrations, and inoculated into TOCs cultured in vitro. There were significant differences in the timing and intensity of the loss of ciliary activity between the TOCs of the different infection groups (*n* = 8). After infection with 10^–4.47^ times the concentration of TOCs in the infection group, more than half of the cilia in the same group stopped proliferating at 7 dpi, and the epithelial cells fell off, while the cilia in the tracheal rings of the blank group were maintained until 8 dpi. Ciliary cessation was observed within 144 h. The summarized results in Table 3 show that the amount of MG inoculated was inversely proportional to the duration of ciliary cessation. The TOC-ID_50_ was calculated according to the Reed–Muench method [27].

**Table 3:** **Summary of cilia cessation by TOC**

Bacterial solution Dilution	The rate of stopping the oscillation of TOC cilia at different times (%)											
	12 h	24 h	36 h	48 h	60 h	72 h	84 h	96 h	108 h	120 h	132 h	144 h
10^–1^	20.83	45.8	62.5	79.16	83.33	87.5	91.2	91.2	95.83	100	100	100
10^–2^	16.66	29.20	45.8	62.5	66.7	66.7	70.83	70.83	75	75	79.2	83
10^–3^	8.33	25	25	29.2	37.7	45.8	45.8	50	58.3	58.3	62.5	66.7
10^–4^	8.33	20.8	20.8	25	29.2	29.2	33.33	37.7	45.8	50	54.2	58.3
10^–5^	4.16	8.33	12.5	20.8	20.8	20.8	25	29.2	29.2	33.33	37.7	41
10^–6^	0	4.16	4.16	8.33	12.5	12.5	20.8	20.8	25	29.2	33.3	37.5
10^–7^	0	0	0	4.16	4.16	8.33	12.5	12.5	20.83	20.83	25	25

The distance ratio was calculated as follows: (percentage of lesions greater than 50–50%)/(percentage of lesions greater than 50–percentage of lesions less than 50%) = (58–50%)/(58–41%) ≈ 0.47.

LgTOC-ID_50_ = Distance ratio × difference between the logarithms of dilutions + logarithm of the dilution with a lesion rate greater than 50% = 0.47 × (−1) + (−4) = − 4.47.

Therefore, the TOC-ID_50_ of this strain was a bacterial suspension diluted to 10^–4.47^/200 μL (Table 4).

**Table 4:** **Determination of the TOC-ID**_**50**_** of MG-HY isolates**

Bacterial solution Dilution	Lesion observation results		Cumulative number of wells		Total number of wells	Grade 3 lesions (%)
	Number of wells with grade 3 lesions	No 3-grade wells	Total number of wells with grade 3 lesions	Number of wells without grade 3 lesions		
10^–1^	8	0	24	0	24	100
10^–2^	7	1	20	4	24	83
10^–3^	6	2	16	8	24	66
10^–4^	5	3	14	11	24	58
10^–5^	4	3	10	14	24	41
10^–6^	4	4	9	12	24	37.5
10^–7^	3	5	6	18	24	25

### In vitro infection model in MG

**Figure 1:**
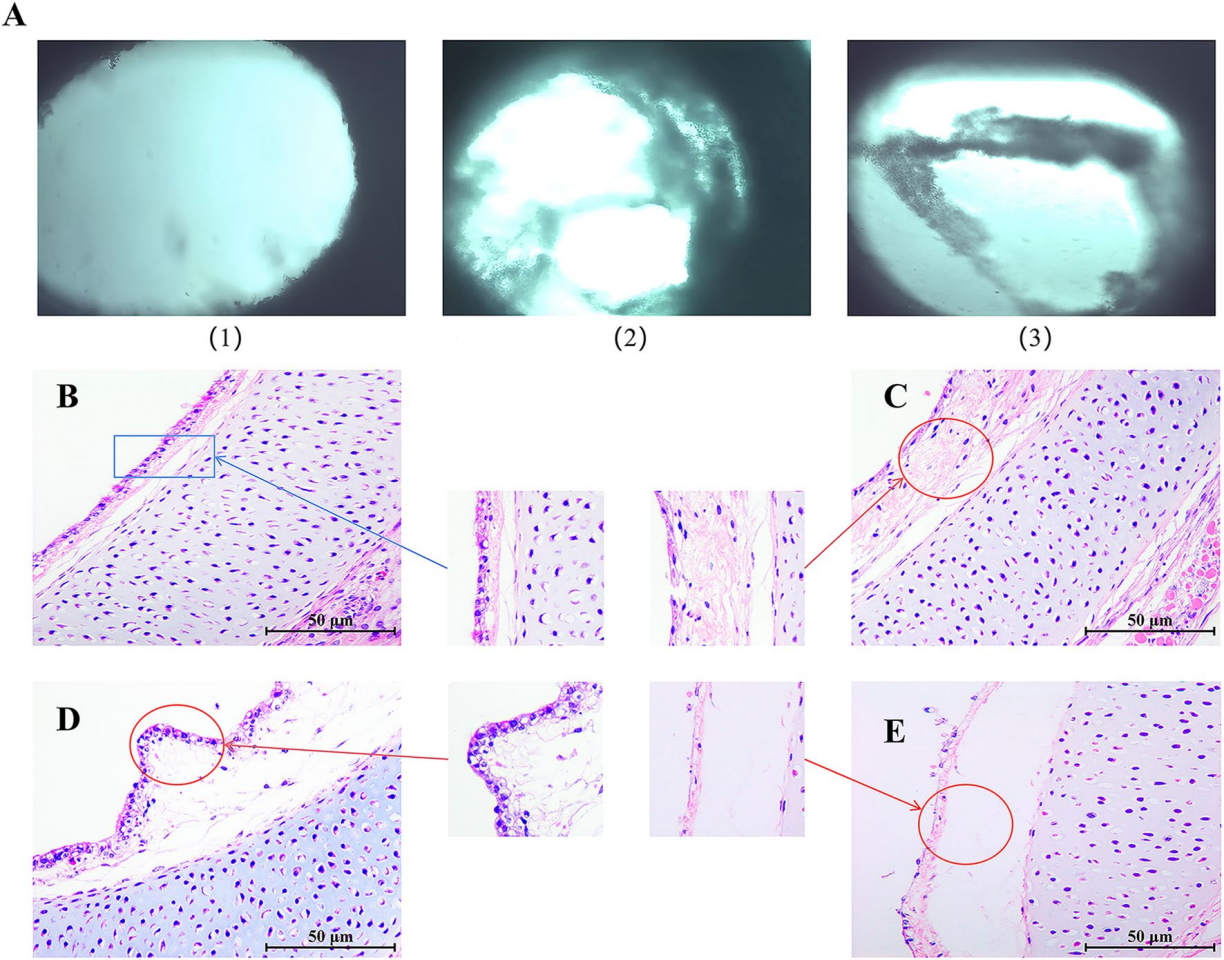
**Comparative morphological changes in chicken embryo tracheal rings in vitro.** (**A**) Microscopic observation (40 ×) of chicken embryo tracheal rings cultured in vitro for 3, 5, 7, and 8 days was performed. (1) In the uninfected group, at 8 dpi, there was normally developed chicken embryo tracheal rings. (2) In the infected group, at 5 dpi, there was atrophy and depression of the tracheal ring epithelial tissue. (3) In the infected group, at 7 dpi, the tracheal ring epithelial tissue was detached. (**B**–**E**) Morphological analysis of chicken embryo tracheal rings was performed by H&E staining. (**B**) A cross-sectional view of the chicken embryo tracheal ring in the uninfected group at 8 days is shown. (**C**) In the infected group, at 3 dpi, the cross-section of the chicken embryo tracheal ring began to degenerate, with mucosal atrophy. (**D**) In the infected group, at 5 dpi, there was further loss of cilia in the cross-section of the chicken embryo tracheal ring, necrosis of the epithelial cells, and the infiltration of inflammatory cells. (**E**) In the infected group, at 7 dpi, there was partial separation of epithelial cells from the lamina propria and detachment and perforation of the epithelial layer.

The TOCs of the selected infection group were exposed to 200 µL/circle (10^–4.47^/200 µL) of MS bacterial solution to establish an infection model, and the TOCs of the control group were continuously cultured in the same amount of PBS. After inoculation with MG solution, the infected TOCs lost their ciliary activity one after another, and at 5 dpi, different degrees of mucosal epithelial abnormalities were observed in all infected groups under inverted microscopy; i.e., large areas of cilia were atrophied or adhered to each other, and even large areas were lost. At 7 dpi, the epithelial surface was significantly eroded, with depressions and cavities (Figure 1A, panels 2 and 3), the uninfected TOCs (8 dpi) had no obvious lesions, and the mucosal surface of the tracheal rings was uniformly flat, with cilia evenly covering their surfaces, while no tissue damage was observed (Figure 1A, Panel 1). Histopathological examination of the chicks revealed that the mucosal structures were intact and that columnar cells were observed in the uninfected group (8 dpi), with no oedema or inflammatory cell infiltration (Figure 1B). Tracheal ciliary motility was maintained for 8 days. In contrast, tracheal epithelial cells in MG-infected chicks began to degenerate at 3 dpi, with loose, atrophied mucosal villi and shedding (Figure 1C). At 5 dpi, the cilia were further shed, resulting in exposure of the lamina propria and necrosis of the epithelium with the infiltration of inflammatory cells (Figure 1D). At 7 dpi, the epithelium was partially separated from the lamina propria and was shed and perforated (Figure 1E).

**Figure 2:**
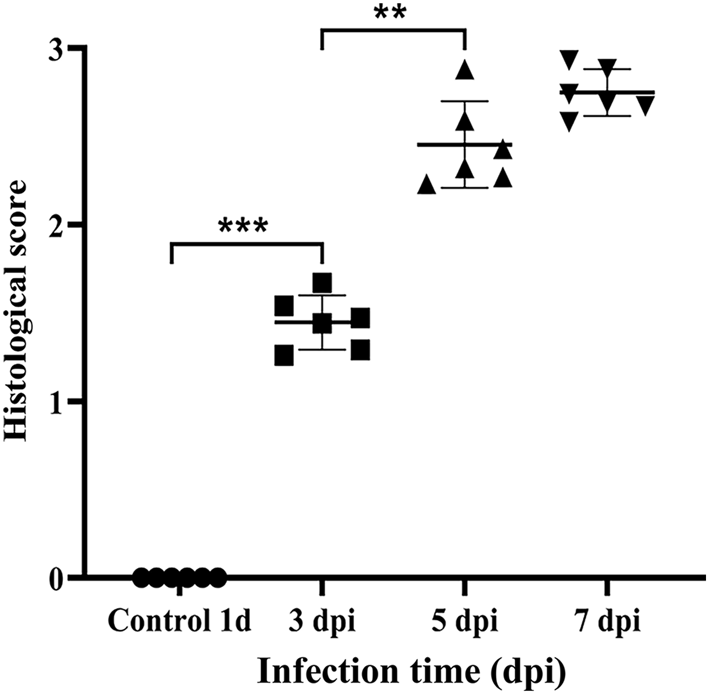
**Scoring of tracheal tissue lesions.** Each point represents a chicken, and the horizontal line represents the average value.

In addition, a thicker intrinsic layer was observed in the MG-infected TOCs than in the mock-infected control TOCs, with histopathological tracheal lesions in MG-infected chickens (*p* < 0.05) (Figure 2). Overall, the results indicated that MG disrupted the morphological structure of the tracheal mucosa and that an MG infection model was successfully established.

### Dynamics of tracheal ring infection

**Figure 3:**
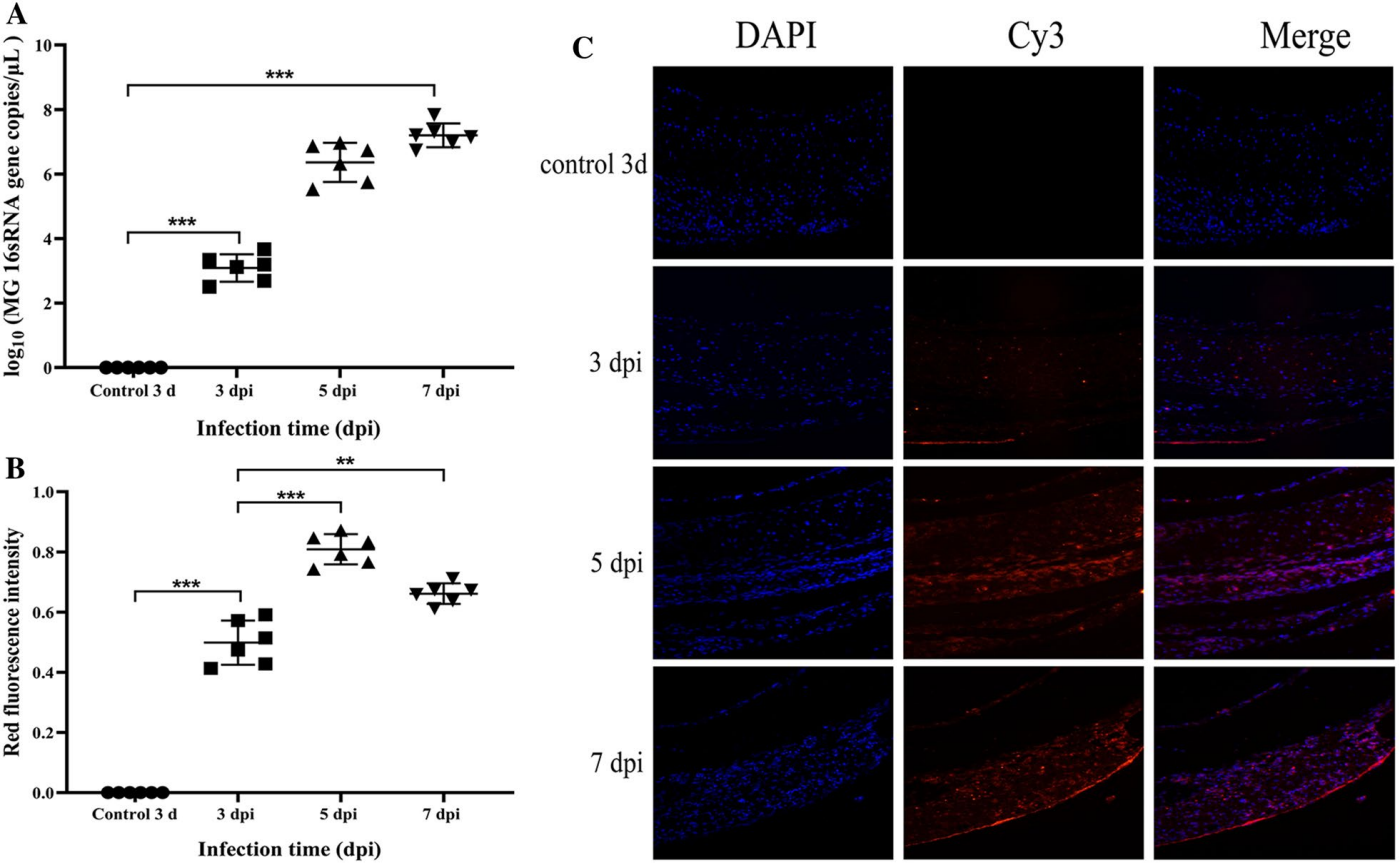
**Kinetics and localization of MG infection in chicken tracheae.** (**A**) Kinetic analysis of TOC exposure to MG was performed. Differences in the bacterial load at 3 dpi, 5 dpi, and 7 dpi are shown, and the fold change was determined via RT‒qPCR. Each point represents a chicken, and the horizontal line represents the average value. (**B**) Quantitative analysis of the intensity of the red fluorescence signal in TOCs infected with MG was performed. (**C**) The specific distribution of MG in tracheal tissue was evaluated by FISH. Fluorescence microscopy was applied to determine the specific distribution of MG in tracheal tissues after infection. The number of bacteria increased significantly with the duration of infection. Nuclei stained with DAPI were blue under UV excitation, positive signals were red for the corresponding fluorescein (CY3) labelling, and mRNA in situ hybridization confirmed that the cytoplasm was theoretically positive, with a few nuclear positives being normal. Micrographs were taken at 400 × magnification (*n* = 8), and the fluorescence brightness was strong or weak depending on the amount of expression.

As assessed by qRT‒PCR, the bacterial load in the tracheal ring tissue increased significantly with prolonged infection relative to that at 3 dpi, and the fastest increase in bacterial gene expression was observed at 5 days, when the expression to reached 10^6^–10^7^ copies/µL; at 7 days, although there was a significant increase, the increase slowed, and the colonization was basically stable at 10^7^ copies/µL (Figure 3A). In addition, FISH experiments using LmCq-specific probes showed a strong MG red fluorescent signal in TOCs (Figure 3B). The mucosa, submucosa, and epithelium of the tracheal rings were densely distributed in the test group. In addition, the red fluorescent signal intensity at 5 and 7 dpi was significantly greater than that at 3 dpi. At 5 dpi, the red fluorescent signal intensity exhibited the greatest increase in intensity and the strongest signal (Figure 3C). The same method was used to analyse the tracheal ring tissue in the control group, but no strong fluorescence signal was detected.

### Effect of MG infection on the mRNA expression of inflammatory cytokines in TOCs

**Figure 4:**
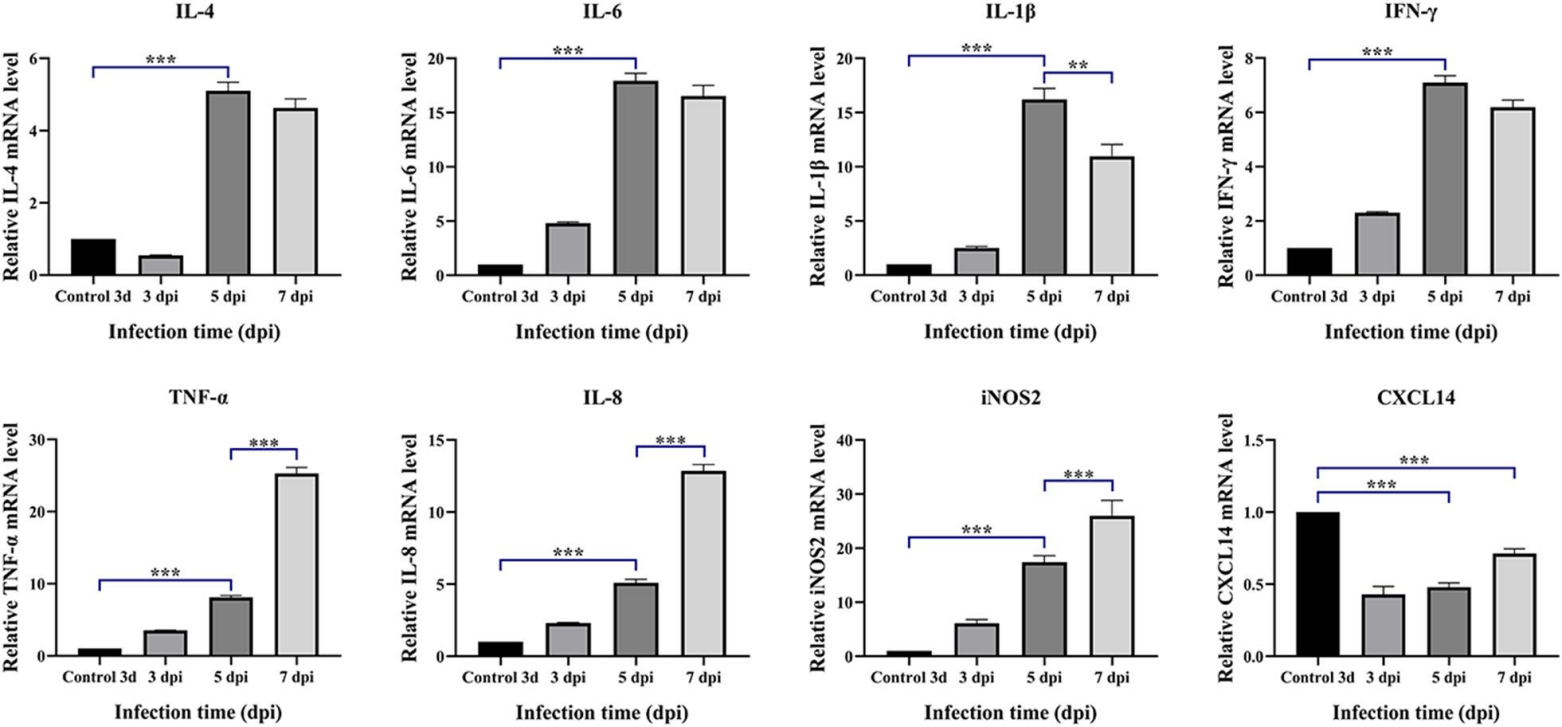
**Effect of MG infection on the mRNA expression of inflammation-related genes in TOCs.** The mRNA expression levels of inflammation-related genes, including TNF-α, IL4, IL6, IL-8, IFN-γ, IL-1β, CXCL14 and iNOS2, were measured.

The effect of exposure to 10^–4.47^ TOCs on inflammatory cytokines was also investigated in the live MG-HY strain. Analysis of the mRNA levels of proinflammatory cytokines revealed that the mRNA expression of all eight genes (IL-4, IL-6, IL-1β, IFN-γ, TNF-α, IL-8, iNOS2, and CXCL14) was upregulated to a much greater extent in the treated group than in the control group. The upregulation of IL4, IL-1β, IL-6, and IFN-γ mRNA expression peaked at 5 dpi compared to those in the control-3d group and diminished thereafter. The TNF-α, IL-8, and iNOS2 levels peaked at 7 dpi (Figure 4) and were significantly greater than those at 5 dpi. Interestingly, we found that CXCL14 mRNA expression was significantly downregulated at the beginning of infection, with a slight recovery at 7 dpi. The stable expression and high abundance of CXCL14, which is a highly active antimicrobial peptide (AMP) that is active against respiratory bacteria, in epithelial tissues contributed to airway bacterial clearance. We hypothesized that CXCL14 helps maintain epithelial tissue homeostasis and exerts sterilizing functions before inflammatory conditions are established rather than being directly involved in immune processes driven by inflammatory responses.

### Effect of MG infection on activation of the NF-κB/MLC2 signalling pathway

**Figure 5:**
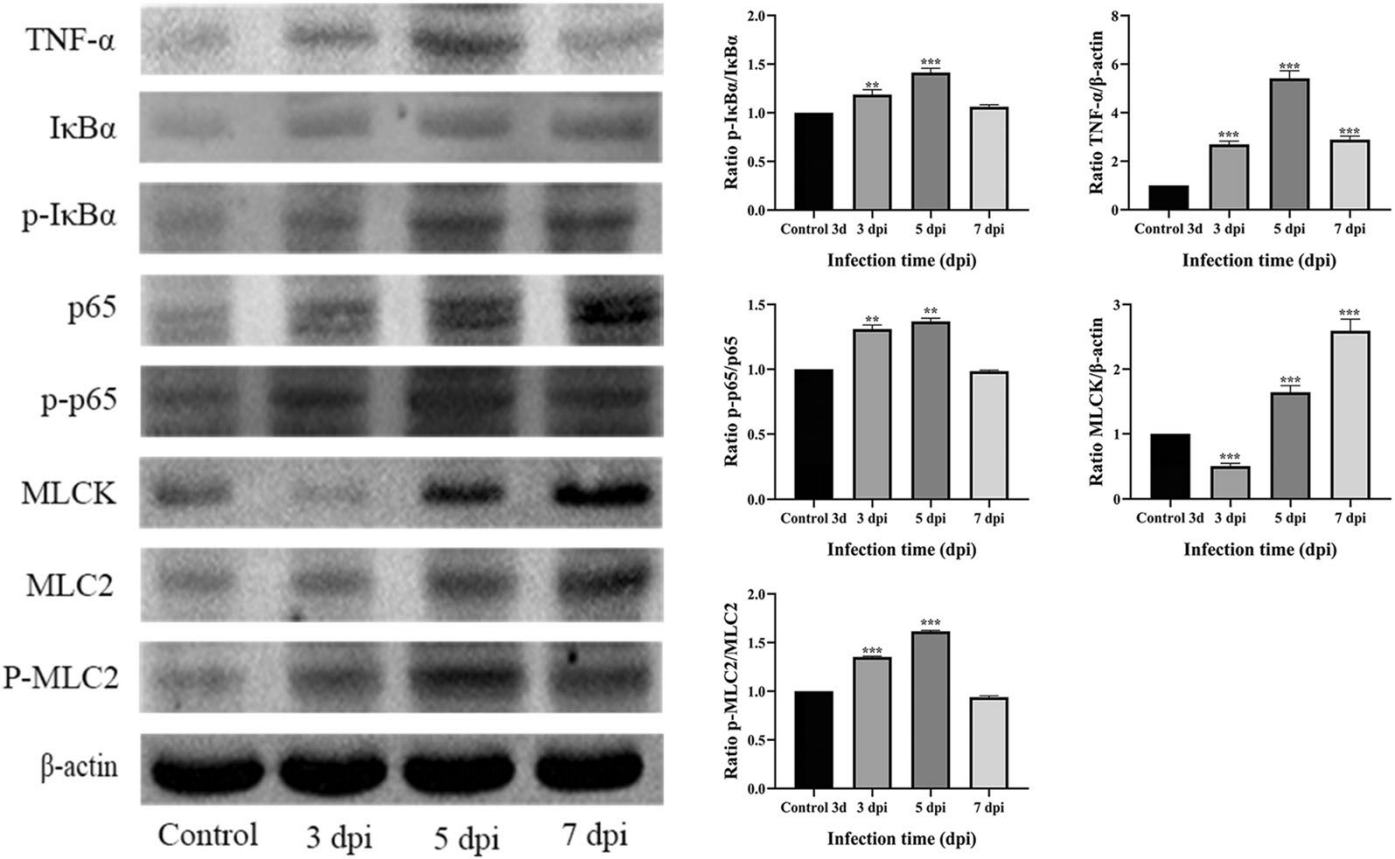
**Western blot analysis of the correlation between the expression of proteins in the NF-κB/MLC2 signalling pathway and total protein expression in TOCs.** Western blotting results showed the protein levels of IκBα, p-IκBα, p65, p-p65, TNF-α, MLCK, MLC2, and P-MLC2. β-Actin was used as an internal control in the experiments. All the bar graphs show the mean ± SD. *p < 0.05; **p < 0.01; ***p < 0.001 represent significant differences compared to the control group.

To further investigate the molecular mechanism of MG-induced tracheal epithelial barrier damage, we analysed the expression of TNF-α-NF-κB/MLCK pathway-related proteins, including IκBα, p-IκBα, p65, p-p65, TNF-α, MLCK, MLC2, and P-MLC2, by protein blotting (*p* < 0.01). Western blotting (Figure 5) showed that, compared with those in the control group, the levels of phosphorylated IκBα and phosphorylated MLC2 in TOC plasma were significantly greater after 5 and 7 days of MG exposure, respectively, and the levels of the NF-κB p65 and MLCK proteins in the nucleus were also significantly higher. Moreover, TNF-α protein expression was significantly increased (*p* < 0.01). These results suggest that MG infection causes inflammatory damage and activates the TNF-α-mediated NF-κB/MLCK signalling pathway from 5 to 7 dpi.

### Effect of MG infection on the mRNA and protein expression of tight junction proteins in TOCs

**Figure 6:**
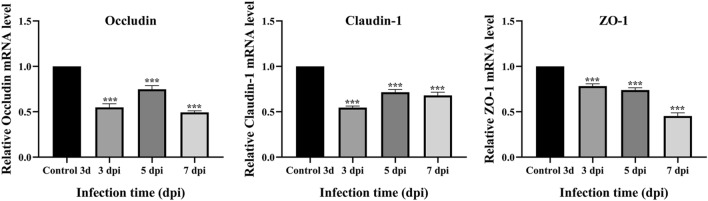
**Relative expression levels of TJ proteins in TOCs infected with MG.** The tight junction-related genes included claudin-1, ZO-1 and occludin.

**Figure 7:**
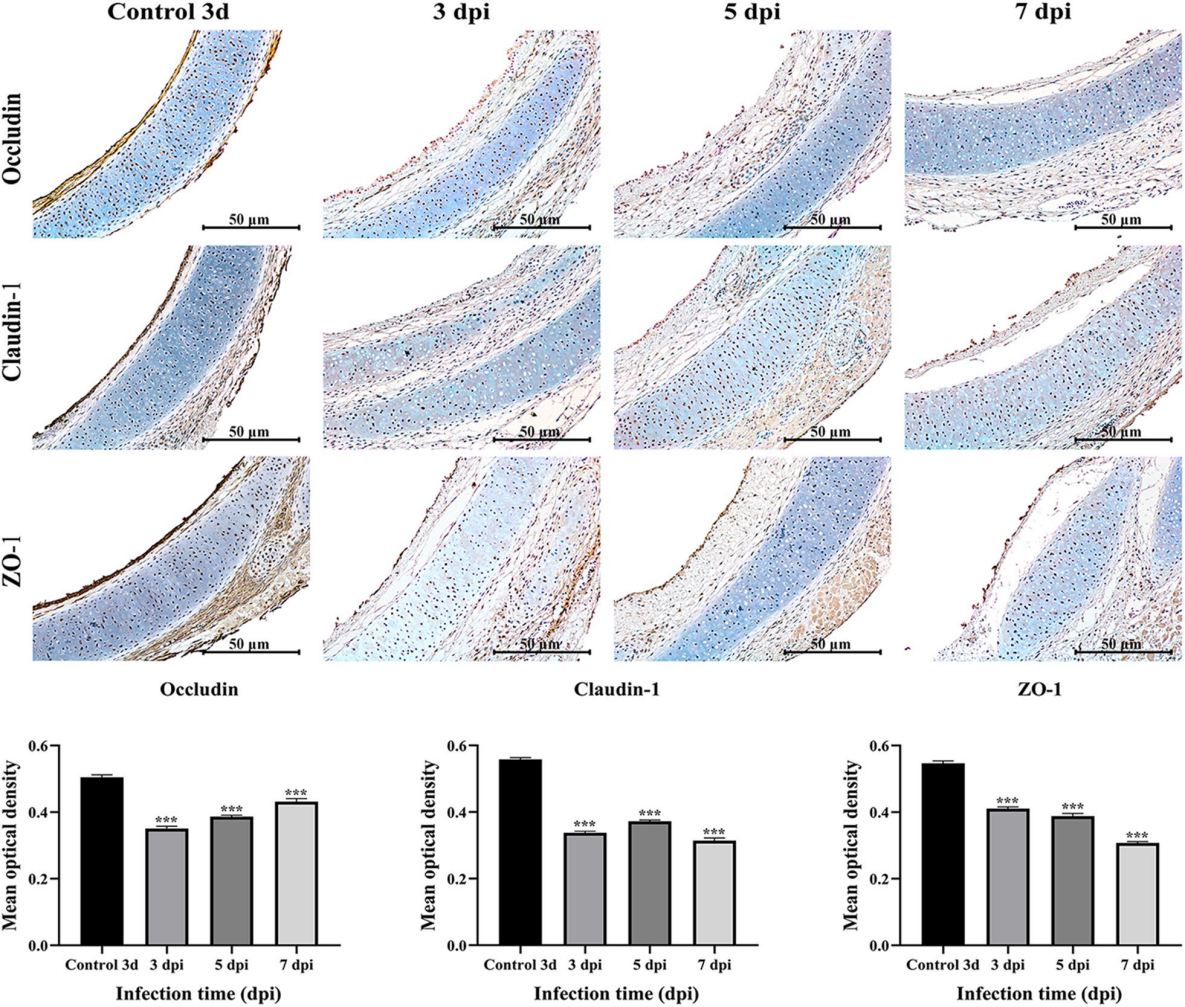
**Immunohistochemical staining of the MG antigen in TOC. The expression and location of claudin-1, ZO-1 and occludin in TOCs were observed via immunohistochemistry.** Claudin-1, ZO-1 and occludin levels in TOCs exposed to MG were determined by densitometry. A significant difference (*p* < 0.01) between the control and MG-treated groups is denoted by an asterisk.

Tight junction (TJ) proteins play a key role in maintaining the tracheal epithelial barrier. Compared with those in the 3 day control group, the mRNA levels of the tight junction-related genes claudin-1, ZO-1 and occludin were significantly lower after TOC was infected with MG, and the mRNA expression levels at each time point were significantly greater than those in the control group (*p* < 0.01) (Figure 6). The expression and localization of claudin-1, ZO-1 and occludin in TOCs were observed via immunohistochemistry (Figure 7). In the control 3d group of TOCs, claudin-1, ZO-1 and occludin were detected in the basal layer of the whole TOC mucosa and outside the tracheal villi. In the MG infection group, the expression of claudin-1 and occludin at 3, 5, and 7 dpi was lower than that in the control group, and the expression of the ZO-1 protein was significantly decreased. The average optical density was quantified via IHC analysis (Figure 7), and the results were consistent with the qRT‒PCR results, indicating that MG infection reduced the expression of TJ proteins in TOCs.

## Discussion

MG infections have been reported on nearly every chicken farm in China. Despite interventions using vaccines and antibiotics, mycoplasma infections continue to spread. This consistent presence of MG increases susceptibility to other diseases, thereby leading to substantial financial losses in the poultry sector [28, 29]. While it is known that MG disrupts the mucosal barrier and leads to tracheal inflammation [12, 30], the exact mechanisms of prolonged MG infection in chickens remain elusive. This knowledge gap hinders a precise understanding of MG-host dynamics.

In the short term, TOC cultivation allows for retention of the normal tissue structure and biological characteristics, facilitating the easier detection of signalling molecules. Moreover, research by *Dykstra* has indicated that in vitro models of avian mycoplasma infection can cause changes in the tracheal ring similar to what is seen in vivo, albeit with more pronounced and earlier cell detachment [31]. Hence, the TOC concentration serves as a valuable tool for studying tracheal mucosal damage caused by infectious agents. In this study, to ensure a consistent bacterial load when inoculating TOC, we adapted a previously described method to establish grading criteria for changes in TOC pathology. Our experimental data revealed that at 10^–4.47^, the bacterial suspension halted the movement of more than half of the cilia within 7 days, leading to epithelial cell detachment in the same group. Therefore, we used a 10^–4.47^/200 μL dilution of the bacterial suspension (TOC-ID_50_) for bacterial challenge in chicks.

The respiratory mucus-ciliary clearance (MCC) function is the main defence mechanism against respiratory pathogens [32]. During this experiment, the results of histopathological examination showed that the tracheal rings infected with MG had varying degrees of mucosal epithelial abnormalities at 5 dpi; that is, large areas of cilia had atrophied, a large area even fell off, and the epithelial surface was obviously eroded, with depression and thickening. Accordingly, we speculate that MG infection leads to the stagnation and loss of cilia and the shedding of epithelial mucosa [33], which may be important causes of secondary infection caused by other pathogens. *Mycoplasma gallisepticum* grew slowly during the first 3 days after infection, and the DNA copy number was only 1 × 10^3.3^ copies/µL. Between 3 and 5 dpi, MG proliferated rapidly to 1 × 10^6.8^ copies/µL in the tracheal ring tissue, and at 7 dpi, although there was a significant increase, the increase slowed, and the amount of colonization was basically stable at 10^7^ copies/µL. Therefore, we speculate that *Mycoplasma gallisepticum* adhered to TOCs may be removed by mucus and cilia in the early stage and that MG can proliferate in TOCs due to the atrophy and shedding of cilia in the later stage. In addition, the content and distribution of *mycoplasma* within the TOC were determined by FISH, which revealed that the red fluorescent signal (MG) detected at 5 dpi was significantly greater than that detected at 7 dpi, indicating that *mycoplasma* did not continuously proliferate, unlike in the MG in the in vivo infection experiments. Wu et al. [34] reported that MG can lead to continuous infection in vivo, and the number of *mycoplasma* species increases with increasing infection time. We suspect that this difference may be related to the use of in vitro culture because MG is a slow-growing pathogenic microorganism that is susceptible to culture conditions. As *mycoplasma* species only have a simplified metabolic pathway, they must obtain essential nutrients from the external environment. When the nutrient content of the medium was limited, replication was difficult. In the present study, as the infection duration of MG increased, the epithelium of the tracheal ring mucosa became infected and shed. At 7 dpi, MG was also observed on the exfoliated tracheal mucosa. The lack of sufficient tracheal mucosa may also explain why MG prevents infection and replication. In vivo, MG can persistently migrate from damaged tracheal mucosa to adjacent healthy tracheal mucosa, resulting in persistent infection and proliferation in the respiratory epithelium of chickens [31, 34]. Therefore, extensive mucosal shedding and the lack of a high-nutrient medium supply may explain why MG could not persist during infection and proliferation in TOCs.

Recent studies have shown that the avian pathogen MG can invade nonphagocytic cells [31, 35]. Furthermore, MG can be cloned within isolated cells and can stably survive and multiply in cells that have been passaged several times in culture media [40]. In addition, Majumder et al. [13, 36] analysed blood samples from chicks infected with the *Mycoplasma gallisepticum* R low strain. *Mycoplasma gallisepticum* R-low was detected in the blood stream of infected chickens, revealing that *mycoplasma* was present not only on the surface but also inside the chicken erythrocytes. Therefore, the invasiveness of MG cells may be an important factor in the systemic transmission of chronic respiratory disease (CRD) in chickens. In MG-infected chickens, MG can be reisolated from internal organs, such as the liver, spleen, brain, kidney, and lymph nodes, although at a lower frequency than from the respiratory tract [37]. The results of the FISH experiments in this study showed that tissue invasion of MG occurred in the TOCs, and MG proliferated heavily in the tissues. However, little is known about the host factors and mechanisms that promote or hinder the adhesion/cell invasion of MG; moreover, the mechanism by which MG crosses the chicken mucosal barrier and migrates from the lumen to tissue cells is unclear.

Previous studies have shown that the mucosal barrier of the trachea separates the lamina propria from the airway and acts as a natural physical barrier against microbial invasion [38]. Selective permeability is achieved by controlling the composition and function of epithelial tight junctions (TJs) [39]. TJs are complexes of multiple proteins, the main members of which are transmembrane proteins, peripheral membrane proteins, and cytoskeletal proteins, such as claudins, ZOs, and occludin proteins [40]. TJs are dynamic structures that play indispensable roles in closing the intercellular space, controlling the transport of substances inside and outside the membrane, and maintaining cell polarity. Specifically, changes in the expression and location of tight junction proteins indicate changes in tight junction permeability. In the present study, the expression of three tight junction proteins (claudin-1, ZO-1 and occludin) was decreased at both the protein and mRNA levels in the infected group. Along with the FISH results, we inferred that the integrity of the tightly connected structures and the increased tissue permeability of the tracheal mucosa were disrupted after MG infection, resulting in MG entering the tissue and proliferating.

Although the underlying mechanism through which MG infection leads to TJ damage has not yet been fully elucidated, some studies have shown a close correlation between the suppression of TJ protein expression and an increase in proinflammatory mediators [41]. Jiang et al. discovered that TNF-α acts as another key cytokine that coordinates tracheal epithelial damage in the context of MG infection and can activate the NF-κB pathway [42]. Growing evidence suggests that when exposed to pathogenic agents, the trachea experiences an increase in intracellular calcium ion levels. This surge prompts myosin light chain kinase (MLCK) phosphorylation of myosin light chain (MLC), a pivotal player in actomyosin dynamics. As a result, the cytoskeletal structure of actomyosin contracts, leading to the disruption of TJs within the tracheal epithelium [43, 44]. Moreover, studies have shown that NF-κB signalling can modulate tracheal epithelial barrier function by upregulating MLCK expression. On another note, in our previous, we research explored the immune-related pathways involved in chicken tracheal damage after MG exposure using RNA-seq technology [45]. The results indicated that post-MG infection, the expression of long-chain MLCK was driven by TNF-α, which further compromised the epithelial cell barrier. TNF-α, by activating NF-κB, resulted in increased permeability of the tracheal epithelial barrier. Hence, these findings led us to question whether MG infection disrupts the mucosal barrier through the TNF-α-NF-κB/MLCK pathway. To this end, we examined the expression levels of genes related to the TNF-α-NF-κB/MLCK pathway. Our data showed that post-MG infection, the expression of inflammatory cytokines such as TNFα, IL-β, and IL-6 was significantly upregulated. p-IκB protein expression was notably higher in the treatment group than in the control group beginning at 6 h after delivery (*p* < 0.05), suggesting that p-IκB induces the degradation of IκBα and promotes the nuclear translocation of NF-κB/p65. The activation of MLCK led to MLC phosphorylation, thereby triggering actomyosin contraction, cytoskeletal remodelling, and a reduction in tight junction protein expression. These results indicate that MG may disrupt TJs by activating the TNF-α-NF-κB/MLCK pathway, resulting in increased epithelial permeability, compromised epithelial barrier integrity, and cilia shedding, which promotes the migration of MG within tissues. Therefore, we speculate that MG might spread through the damaged airway epithelium from the trachea, but further research is needed to confirm this hypothesis.

Clinical studies have shown [46, 47] that by inhibiting NF-κB/p65 activation through pharmacological inhibition or siRNA-mediated silencing of the p65 subunit, TNF-α-induced MLCK gene activation and the increase in TJ permeability can be completely suppressed. However, whether TNF-α blockers reduce airway epithelial damage caused by increased TJ permeability has not yet been determined. Although there is a lack of related research, these findings suggest the potential usefulness of TNF-α inhibitors for preventing and treating airway mucosal damage caused by MG infection in chickens. Considering the significance of TOC as an in vitro model and the potential implications of our current findings, we plan to further validate these observations in subsequent experiments and investigate the therapeutic efficacy of TNF-α inhibitors for airway mucosal damage caused by MG infection in chickens.

In summary, the results of this study provide significant new insights into the signalling process by which MG mediates the regulation of tracheal epithelial TJ permeability via TNF-α in an in vitro chicken embryo trachea model. The data suggest a potential association between the increase in tracheal TJ permeability induced by TNF-α and activation of the MLCK gene, possibly involving the TNF-α-NF-κB/MLCK axis. This research provides insight into the molecular mechanisms of MG infection and potential pathways involved in persistent in vivo infection by *M. gallisepticum*.

## Data Availability

All data generated or analyzed during this study are included in this published article and its supplementary information files. These data encompass, but are not limited to, raw experimental data, statistical analyses, and additional charts or datasets that support the findings of this study. These materials provide support for the transparency and reproducibility of the research and are available for peer review and public inquiry. For any further inquiries regarding the data, we welcome fellow researchers to contact the corresponding author at [fafuwyj@163.com]. We are committed to providing additional information and data support within reasonable bounds.

## Abbreviations

MG: *Mycoplasma gallisepticum*
TOC: tracheal organ culture
CRD: chronic respiratory disease
MLC: myosin light chain
MLCK: myosin light chain kinase
FBS: fetal bovine serum
NAD: nicotinamide adenine dinucleotide
H&E: haematoxylin and eosin
DAB: diaminobenzidine
IOD: integrated optical density
dpi: days post-infection
AMP: antimicrobial peptide
TJ: tight junction
